# Early death after palliative radiation treatment: 30-, 35- and 40-day mortality data and statistically robust predictors

**DOI:** 10.1186/s13014-023-02253-0

**Published:** 2023-04-03

**Authors:** Carsten Nieder, Luka Stanisavljevic, Bård Mannsåker, Ellinor C. Haukland

**Affiliations:** 1 Department of Oncology and Palliative Medicine, Nordland Hospital Trust, 8092 Bodø, Norway; 2 Department of Clinical Medicine, Faculty of Health Sciences, UiT – The Arctic University of Norway, Tromsø, Norway; 3 Department of Quality and Health Technology, SHARE – Center for Resilience in Healthcare, Faculty of Health Sciences, University of Stavanger, Stavanger, Norway

**Keywords:** Radiation therapy, Bone metastases, Palliative treatment, Prognostic factors, Fractionation

## Abstract

**Background:**

This study analyzed mortality after radiotherapy for bone metastases (287 courses). Endpoints such as treatment in the last month of life and death within 30, 35 and 40 days from start of radiotherapy were evaluated.

**Methods:**

Different baseline parameters including but not limited to blood test results and patterns of metastases were assessed for association with early death. After univariate analyses, multi-nominal logistic regression was employed.

**Results:**

Of 287 treatment courses, 42 (15%) took place in the last month of life. Mortality from start of radiotherapy was 13% (30-day), 15% (35-day) and 18% (40-day), respectively. We identified three significant predictors of 30-day mortality (performance status (≤ 50, 60–70, 80–100), weight loss of at least 10% within 6 months (yes/no), pleural effusion (present/absent)) and employed these to construct a predictive model with 5 strata and mortality rates of 0–75%. All predictors of 30-day mortality were also associated with both, 35- and 40-day mortality.

**Conclusion:**

Early death was not limited to the first 30 days after start of radiotherapy. For different cut-off points, similar predictive factors emerged. A model based on three robust predictors was developed.

## Introduction

Topics such as value-based care, quality-of-care indicators, cost-effectiveness and overtreatment have received considerable attention in the oncological literature [1]. Special consideration is necessary in the palliative and terminal phase of anti-cancer treatment, where mismatch between side effects, cost and other disadvantages of interventions on one hand and expected benefit on the other hand should be minimized. Among factors to consider are an intervention’s aim, e.g., life-prolonging versus symptom-directed, and time-frame aspects such as remaining life time and duration of treatment. Palliative radiotherapy is among the most effective and cost-effective interventions and can be tailored to individual patients’ need and preferences [2–5]. Extreme hypofractionation cuts treatment duration into a fraction of what is needed to complete traditional regimens, e.g. 3 Gy × 10 [6]. As suggested by a recent large meta-analysis [7], there is room for improvement of physicians’ prescription habits or ability to decipher prognosis, because the authors found that 16% of patients with advanced cancer receiving palliative radiotherapy died within 30 days of treatment. In other words, the remaining life time with, e.g. reduced pain if this was the goal of treatment, may have been too short to outweigh the burden or side effects of radiotherapy in a proportion of patients. Typically, decision regret analyses are not performed near the end of life and it is thus difficult to estimate how many patients would have consented to radiotherapy in the final phase of cancer progression, had they been able to judge outcomes in advance.

There are different ways of measuring radiotherapy utilization near the end of life, e.g. 30-day mortality calculated from start of treatment, 30-day mortality calculated from end of treatment, or treatment in the last 30 days of life. In addition, one might be tempted to ask why radiotherapy performed, e.g., in the last 28 days of life is fundamentally different from treatment one or two weeks later. Does the arbitrary 30-day cut-off represent a sound definition, because the early death rate is highest, e.g., at 20–30 days and patients living beyond that mark often survive for another 2–3 months? Or is death a continuous event necessitating a broader evaluation of alternative time frames? In principle, a peak might exist just outside the 30-day time period. These considerations and open questions led us to study death rates and predictors of 30-, 35- and 40-day mortality in an already established database with many baseline parameters that are lacking in large registries such as the National Cancer Database (NCDB) or the Surveillance, Epidemiology, and End Results (SEER) program.

## Patients and methods

Our single-institution database (2014–2019) includes 219 consecutive patients with bone metastases managed with standard palliative external beam radiotherapy regimens such as a single fraction of 8 Gy, 5 fractions of 4 Gy or 10 fractions of 3 Gy (3-D conformal or intensity-modulated; no stereotactic ablative body radiotherapy). Fractionation was at the discretion of the treating oncologist. Additional lesions were treated as indicated, e.g., soft tissue or lung metastases. In other words, a proportion of patients received radiotherapy to several target volumes at the same time. Interrupted or permanently discontinued radiotherapy series were included to comply with the intention-to-treat principle. Standard-of-care systemic anticancer treatment was given as indicated (tailored to organ function, frailty etc.). Patients who returned for a new treatment course (re-irradiation or new target volume) in the time period of the study were counted twice, resulting in a total number of 287 evaluable treatment courses. In returning patients, actual blood test results, imaging reports, Karnofsky performance status (KPS), weight and other baseline data, as well as survival were registered for each individual treatment course. Imaging and blood tests were part of standard oncological assessment and typically no older than 3 weeks before radiotherapy. Most patients had blood tests taken at the day of treatment planning. All blood test results were dichotomized (normal/abnormal) according to the institutional upper and lower limits of normal.

The review-board approved database is regularly updated for survival and has been utilized for different quality-of-care projects before [8, 9]. Overall survival (time to death) from the first day of radiotherapy was calculated employing the Kaplan–Meier method for all 287 treatment courses (SPSS 28, IBM Corp., Armonk, NY, USA). In 27 cases, survival was censored after median 36 months of follow-up (minimum 28 months). Outcomes of interest (30-, 35-, 40-day mortality from start; death within 30 days of last radiation treatment) were dichotomized (alive/dead) and the chi-square test (2-sided) was utilized for further analyses. A multi-nominal logistic regression analysis was also employed. *P*-values ≤ 0.05 were considered statistically significant. The methods employed by Rades et al. were utilized to calculate a point sum reflective of 30-day mortality [10, 11]. For example, a risk factor associated with 50% 30-day mortality was assigned 5 points, while 3 points were assigned for a factor associated with 30% 30-day mortality.

## Results

**Fig. 1:**
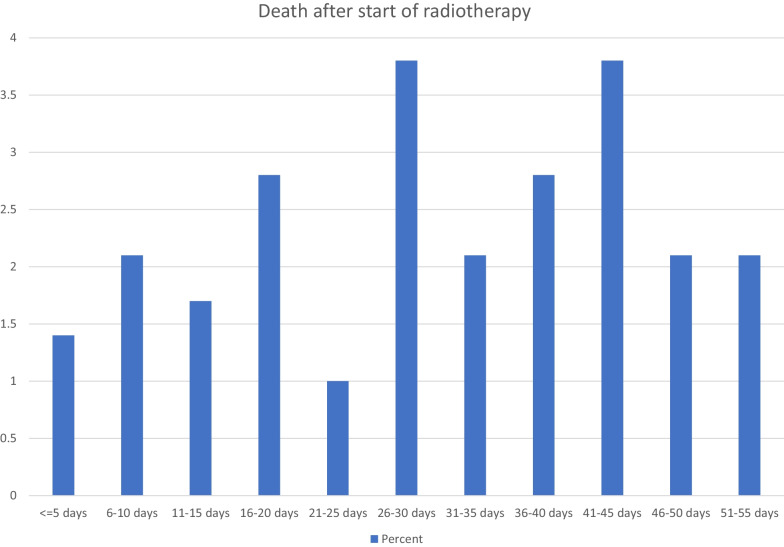
Proportion of treatment courses where patients died shortly after start of palliative radiotherapy for bone metastases (5-day intervals)

**Fig. 2:**
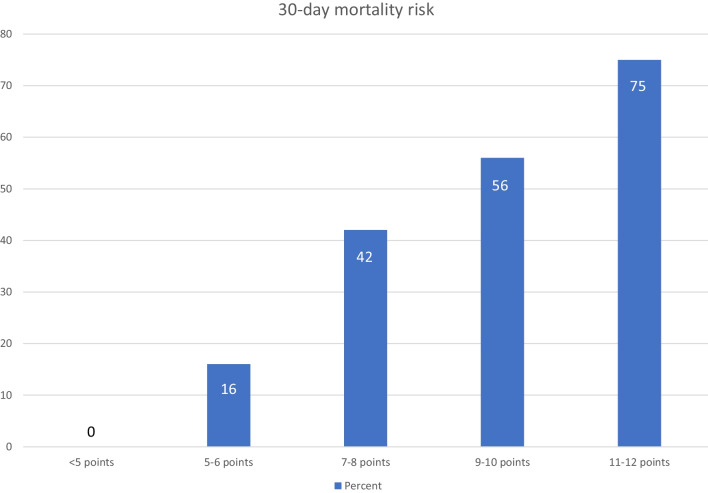
Outcomes of the 30-day mortality risk prediction model (3 variables, as shown in Table 3)

Regarding all 287 treatment courses, 42 (15%) took place in the last month of life. Mortality from start of radiotherapy was 13% (30-day), 15% (35-day) and 18% (40-day), respectively. As indicated in Fig. 1, the 30-day landmark is not particularly representative for early death. Death rates were lower in the first 15 days and increased between day 16 and 45. None of the 5-day intervals can be characterized as outlier. Median actuarial overall survival was 6 months (1-year rate 32%). Table 1 describes the patient-, tumor- and treatment-related baseline characteristics. The impact of all these baseline characteristics on 30-, 35- and 40-day mortality was examined and Table 2 shows that a large number of significant correlations was identified in univariate analyses. All predictors of 30-day mortality were also associated with both, 35- and 40-day mortality. Predictors with *p* ≤ 0.05 in univariate analyses were included in multi-nominal logistic regression analyses. The one for 30-day mortality confirmed KPS (≤ 50 with hazard ratio (HR) 3.7 and 60–70 with HR 1.8, *p* < 0.001), weight loss (HR 1.8, *p* = 0.01) and presence of pleural effusion (HR 7.5, *p* = 0.006) as independent predictors, whereas, e.g., cancer type, blood test results and treatment-related parameters lost their significance. All three significant predictors of 30-day mortality maintained their impact in an exploratory analysis of 40-day mortality with *p* = 0.001–0.003. Additional predictors emerged, albeit with different *p*-values. These included adrenal gland metastases (*p* = 0.02), progressive disease outside of the irradiated region(s) (*p* = 0.03), and serum creatinine (normal versus abnormal, *p* = 0.02). Interestingly, all three additional predictors were also identified in the earlier analyses displayed in Table 2 (bold text), because of disproportional increase of % mortality over time. Finally, the three significant predictors of 30-day mortality were employed to construct a predictive model based on the methodology developed by Rades et al. [10, 11]. Table 3 shows how the point sum can be calculated and Fig. 2 displays the corresponding 30-day mortality rates of 0–75%.

**Table 1:** Baseline characteristics for 219 patients managed with 287 treatment courses. Information is displayed for individual treatment courses

Baseline parameter	Number	Percent
Female sex	118	41
Male sex	169	59
Age ≤ 70 years	154	54
Age 71–80 years	94	33
Age ≥ 81 years	39	14
Prostate cancer	72	25
Non-small cell lung cancer	56	20
Breast cancer	53	19
Small cell lung cancer	11	4
Renal cell cancer	17	6
Colorectal cancer	32	11
Bladder cancer	10	4
Other primary tumors	36	12
KPS < 50*	22	8
KPS 50–70*	153	53
KPS 80–100*	112	39
Outpatient**	182	63
Inpatient**	105	37
One or two target volumes irradiated	206	72
Three or more target volumes irradiated	81	28
Osseous metastases irradiated (exclusively)	234	82
Extraosseous metastases irradiated	53	18
Pain indication for RT	245	85
Non-pain indication (neurological etc.)	42	15
Prescribed regimen of 10 fractions	100	35
Prescribed regimen of 1 fraction	70	24
Prescribed regimen of 2–5 fractions	117	41
Incomplete radiotherapy	9	3
No systemic therapy	64	22
Ongoing systemic therapy within last 4 weeks	180	63
Systemic therapy > 4 weeks before RT	43	15
Systemic chemotherapy	99	34
Endocrine treatment	101	35
Immune checkpoint inhibitor	9	3
Tyrosine kinase inhibitor	14	5
Corticosteroid concomitant to RT	115	40
No corticosteroid concomitant to RT	172	60
Opioid analgesic concomitant to RT	189	66
No opioid analgesic concomitant to RT	98	34
Palliative care team involved**	96	33
Palliative care team not involved**	191	67
Early RT, within 2 mo from cancer diagnosis	91	32
Late RT, > 2 months	196	68
Low hemoglobin*	174	61
Normal hemoglobin*	112	39
Hypercalcemia*	18	6
Normal calcium*	262	91
Low albumin*	41	14
Normal albumin*	229	80
High lactate dehydrogenase*	116	40
Normal lactate dehydrogenase*	122	43
High alkaline phosphatase*	157	55
Normal alkaline phosphatase*	111	39
Leukocytosis*	54	19
Leukopenia*	13	5
Normal leukocytes*	219	76
High C-reactive protein > 90 mg/L*	39	14
High C-reactive protein < 90 mg/L*	159	55
Normal C-reactive protein*	84	29
Abnormal platelet count*	56	20
Normal platelet count*	229	80
Low creatinine*	61	21
Normal creatinine*	194	68
High creatinine*	32	11
Brain metastases	25	9
Liver metastases	87	30
Lung metastases	93	32
Adrenal gland metastases	23	8
Pleural effusion***	27	9
Disease progression in non-irradiated area	132	46
Stable disease outside irradiated area	152	53
Weight loss of at least 10% (last 6 mo)	98	34

*KPS* Karnofsky performance status, *RT* radiotherapy

*At treatment planning, typically one week before start of treatment

**At start of treatment

***Regardless of symptoms and amount; identified on treatment planning computed tomography or the preceding diagnostic chest imaging (any type)

**Table 2:** Significant predictors of short survival from start of radiotherapy

Parameter	% 30-day mortality	% 35-day mortality	% 40-day mortality	Significance level*
KPS 30	75	81	81	< 0.001 (30, 35, 40)
KPS 40	33	33	33	
**KPS 50**	**57**	**64**	**79**	
KPS 60	14	18	22	
KPS 70	9	11	14	
KPS 80	0	0	2	
KPS 90–100	0	0	0	
Inpatient	26	30	33	< 0.001 (30, 35, 40)
Outpatient	5	7	9	
Weight loss of at least 10% (6 mo)	28	31	37	< 0.001 (30, 35, 40)
Stable weight	1	1	3	
Prostate cancer	7	8	8	0.03, 0.04, 0.05
Breast cancer	4	8	9	
Small cell lung cancer	9	9	18	
Non-small cell lung cancer	20	21	27	
Colorectal cancer	16	19	19	
Bladder cancer	30	30	30	
Renal cell cancer	35	35	41	
**Other cancer**	**8**	**14**	**19**	
No systemic treatment	22	25	28	0.004, 0.005, 0.001
Systemic treatment last 4 weeks	7	9	11	
Systemic treatment > 4 weeks before RT	23	26	30	
Chemotherapy	15	18	23	0.02, 0.02, 0.01
Endocrine therapy	4	5	6	
Immune checkpoint inhibitor	11	11	11	
Tyrosine kinase inhibitor	21	21	21	
Lactate dehydrogenase high	20	21	24	0.01, 0.05, 0.02
Lactate dehydrogenase normal	8	11	12	
Albumin low	39	44	46	< 0.001 (30, 35, 40)
Albumin normal	8	10	13	
Hemoglobin low	19	22	26	< 0.001 (30, 35, 40)
Hemoglobin normal	4	4	5	
Leukocytes low	23	23	23	0.001, 0.001, 0.002
Leukocytes normal	9	11	14	
Leukocytes high	26	30	33	
**Creatinine low**	**28**	**31**	**38**	< 0.001 (30, 35, 40)
Creatinine normal	6	8	10	
Creatinine high	25	28	28	
CRP > 90 mg/L	38	44	46	< 0.001 (30, 35, 40)
CRP 5–90 mg/L	13	15	19	
CRP normal	2	2	4	
Brain metastases	32	32	36	0.008, 0.03, 0.02
No brain metastases	11	13	16	
Liver metastases	20	22	25	0.04, 0.05, 0.04
No liver metastases	10	12	15	
**Adrenal gland metastases**	**30**	**35**	**43**	0.02, 0.01, 0.002
No adrenal gland metastases	11	13	16	
**Pleural effusion**	**33**	**41**	**44**	0.002, < 0.001, < 0.001
No pleural effusion	10	11	14	
**Disease progression outside RT area**	**23**	**27**	**33**	< 0.001 (30, 35, 40)
No progression outside RT area	3 ara>	4	5	
Opioid analgesics	20	22	25	< 0.001 (30, 35, 40)
No opioid analgesics	0	1	3	
Steroid medication	23	27	30	< 0.001 (30, 35, 40)
No steroid medication	6	7	10	
Palliative care team involved	24	29	31	< 0.001 (30, 35, 40)
Palliative care team not involved	7	8	11	

Bold: at least 10% longitudinal increase between 30 and 40 days

*KPS* Karnofsky performance status, *RT* radiotherapy, *CRP* C-reactive protein

*As predictive factor for each 30-, 35- and 40-day mortality; over all strata, e.g. for KPS or cancer type

**Table 3:** Final 30-day mortality prediction model

Parameter	Points	% 30-day mortality
Stable weight	0	1
Weight loss of at least 10% (6 mo)	3	28
KPS 80–100	0	0
KPS 60–70	1	11
KPS ≤ 50	6	61
No pleural effusion	1	10
Pleural effusion	3	33

*KPS* Karnofsky performance status

## Discussion

This study compared death rates during different time intervals in the early phase after radiotherapy and identified variables that impact on, e.g., 30-day mortality. Early death was not limited to the first 30 days after start of radiotherapy. Relatively similar death rates were seen between day 16 and 45. Focusing on 30-day mortality, a widely used endpoint in the literature (radiotherapy and other approaches), is thus an arbitrary decision (some sort of cut-off is needed) and not necessarily data-driven, as shown in the present example. Furthermore, palliative radiotherapy is not normally associated with procedure-related mortality, in contrast to, e.g. surgery. The present results also demonstrate that mortality rates depend on the method of evaluation. 30-day mortality from start of treatment was 13%, while 15% of courses were administered in the last 30 days of life. Modest increase of the cut-off, from 30 to 40 days from start of radiotherapy, increased the rate from 13 to 18%. In a recent large meta-analysis [7], 16% of patients with advanced cancer who had received palliative radiotherapy died within 30 days of treatment.

In contrast to several previous studies, the present one included an unusually large number of baseline parameters, both traditional predictors of survival such as KPS, and less well-studied variables such as presence of pleural effusion and numerous blood test results. All predictors of 30-day mortality were also associated with both, 35- and 40-day mortality, and thus robust. Nevertheless, with increasing time interval and number of events (higher death rate after 40 days), additional predictors of early death emerged, albeit with clearly different *p*-values. These dynamics suggest that an increasing number of co-variates impact on death rates in analyses that cover a longer time period. KPS, weight loss and pleural effusion maintained their highly significant role and were therefore employed to construct a predictive model, which performed well (Fig. 2). Pleural effusion, which was observed, e.g., in patients with lung, breast and prostate cancer, was not necessarily symptomatic and did not always necessitate intervention. Our study did not include patient-reported dyspnea, which in previous studies was associated with poor prognosis [12]. These two factors might be interrelated, an issue that can only be clarified in prospective studies.

The present results are in line with numerous prognostic models that include KPS as a main and indisputable driver of poor prognosis [13–16]. However, additional factors are important to fully elucidate the likelihood of survival at different time points. Their role requires further study in larger databases. Besides number of patients, limitations of the present work include its retrospective single-institution design and selection bias, because a proportion of poor-prognosis patients referred to palliative radiotherapy might die already before planned start. On the other hand, the study cohort represents a real-world patient population of often elderly patients with highly variable disease burden and survival. Furthermore, we had access to a broad set of baseline parameters and were therefore able to extend the knowledge provided by previous, otherwise similar studies. Patients with brain metastases, a small subgroup in the present study, might represent a special population, if treated to the brain [17] rather than skeletal metastases after previous brain-directed therapy. Our group’s previous work [17] resulted in different predictors of 30-day mortality after treatment for brain metastases (n = 100 patients) than those identified in the present bone metastases study, e.g., number of brain metastases and primary tumor control.

Despite progress in prognostic stratification, survival predictions in oncology tend to be overly optimistic [18–20]. Not all patients initially thought to represent suitable candidates for radiotherapy are able to complete their treatment. In a recent study by Vázquez et al., 30-day mortality after palliative radiotherapy was 17.5% [21]. In the multivariate analysis, male gender, ECOG PS 2–3, gastrointestinal and lung cancer were found to be independent factors related to this endpoint. Weight loss and other parameters available in the present study were not included. The large meta-analysis by Kutzko et al. identified multiple treatment sites, hepatobiliary primary, inpatient status, and ECOG PS 3–4 as predictors of 30-day mortality [7]. In contrast to these results, Wu et al. performed a multivariate analysis suggesting that breast or prostate primary tumor, ECOG PS, body mass index, liver metastases, more than 5 active metastases (dichotomized, radiographically identified), albumin level, and hospitalization within 3 months of radiotherapy consult were associated with 30-day survival [19]. Harmonization efforts and cooperation are needed to arrive at generally accepted and widely implemented predictive models, or a single consensus model. So far, it seems that PS and primary tumor type are common and well-established predictors, while contradictory results were obtained for other variables. Ideally, prospective comparisons should be attempted to clarify the role of potentially redundant variables such as patient-reported dyspnea, radiological presence of lung metastases or pleural effusion, and blood test results such as anemia, which might impact on dyspnea.

Patients at high risk of early death should preferably be managed with single-fraction radiotherapy for bone metastases [22, 23], if they prefer radiotherapy over other palliative and supportive measures aiming at pain control. Even patients with longer survival can often achieve satisfactory pain control with such simple treatment, if uncomplicated bone metastases are present, and sometimes additional re-irradiation is able to “boost” and prolong the effect of initial treatment. Special scenarios such as impending fractures, post-operative radiotherapy, large extra-osseous infiltration or ablation of oligometastases require thorough assessment of advantages and disadvantages of prolonged courses of radiotherapy or stereotactic body radiotherapy.

## Conclusions

Early death was not limited to the first 30 days after start of palliative radiotherapy for bone metastases. For different cut-off points (30-, 35-, 40-day mortality), similar predictive factors emerged. A model based on three robust predictors was developed, which is easily applicable in clinical practice. External validation by other institutions is warranted.

## Data Availability

The datasets used and/or analyzed during the current study are available from the corresponding author on reasonable request.
